# Protective Equipment in Football: A Review of History, Evolution, Materials, and Contemporary Use

**DOI:** 10.3390/sports13110392

**Published:** 2025-11-05

**Authors:** Marco Vecchiato, Luca Russo, Alberto Livio, Emanuele Zanardo, Mara Mezzalira, Emanuele Farina, Andrea Demeco, Stefano Palermi

**Affiliations:** 1 Sports and Exercise Medicine Division, Department of Medicine, University of Padova, Via Giustiniani 2, 35128 Padova, Italy; alberto.livio2@gmail.com (A.L.); ema_zanardo@hotmail.it (E.Z.); 2 Department of Theoretical and Applied Sciences, eCampus University, 22060 Novedrate, Italy; 3 Corte Medica, Via Molino 27, 36027 Rosà, Italy; 4 Physical and Rehabilitative Medicine, Department of Medical and Surgical Sciences, University of Catanzaro “Magna Graecia”, 88100 Catanzaro, Italy; andreademeco@hotmail.it; 5 Department of Medicine and Surgery, UniCamillus-Saint Camillus International University of Health Sciences, 00187 Rome, Italy; stefano.palermi@unicamillus.org

**Keywords:** protective equipment, soccer, injury prevention, face mask, sports biomechanics, safety

## Abstract

Football (soccer) is the world’s most widely played sport, but it carries a high incidence of traumatic injuries, particularly to the head, face, and lower limbs. Once regarded as a low-equipment discipline, the role of protective devices has expanded substantially in recent decades, both in injury prevention and in return-to-play strategies. This review provides a comprehensive overview of the historical evolution, typology, and materials of football protective equipment, with additional focus on regulatory frameworks, cultural acceptance, and illustrative cases from elite athletes. Shin guards remain the only mandatory device, yet the use of facial masks, headgear, braces, and orthoses is increasing, particularly following high-profile injuries. Advances in carbon fiber composites, thermoplastics, viscoelastic foams, and additive manufacturing have enabled lightweight, customized devices that balance protection with comfort and adherence. Beyond biomechanics, psychological reassurance, esthetics, durability, and hygiene strongly influence player compliance and perception. Despite this progress, critical challenges remain. Football lacks standardized testing protocols, clear certification pathways, and longitudinal studies on long-term outcomes. Evidence is particularly limited for youth athletes and newer categories of equipment. Looking ahead, the integration of wearable technologies, systematic hygiene and durability testing, and sustainable materials could transform protective gear into multifunctional tools for safety, monitoring, and performance optimization. Protective equipment in football has thus evolved into a multidisciplinary field at the intersection of medicine, engineering, psychology, and regulation. Future advances will depend on stronger collaboration between clinicians, researchers, governing bodies, and manufacturers to ensure safe, effective, and widely accepted protective solutions at all levels of the game.

## 1. Introduction

Football (also known as soccer) is the most popular sport worldwide, played by over 250 million athletes across all age groups [1]. While it is often celebrated for its dynamism, technical skill, and accessibility, football is also characterized by frequent physical contact, high-speed actions, and unpredictable collisions, all of which contribute to a significant risk of injury [2,3]. Injuries most frequently involve the lower extremities [4], but head and facial traumas also represent a substantial clinical concern, often leading to significant time-loss and long-term sequelae. Recent epidemiological studies have shown that football matches have injury incidence rates of approximately 20 injuries per 1000 h of exposure, whereas training injury rates are substantially lower at around 2–3 injuries per 1000 h [5,6]. Although traditionally considered a low-equipment sport, these data highlight the urgent need for standardized protective strategies and objective evaluation methods for football equipment, comparable to those adopted in other contact sports [7,8].

Protective equipment in football serves primarily to reduce injury risk and to support safe return-to-play. It includes mandatory gear (shin guards), optional but referee-approved devices (e.g., masks, headgear, goggles), and auxiliary supports (e.g., braces, taping), typically manufactured with materials such as thermoplastics, carbon fiber, and viscoelastic foams. The integration of materials science and biomechanics has been pivotal in driving these innovations, allowing not only enhanced impact resistance but also improved comfort, durability, and player adherence. In the early days of football, protective equipment was scarce and often improvised [9]. A major change came in 1990 when Fédération Internationale de Football Association (FIFA) made shin guards mandatory, which paved the way for the gradual introduction of other specialized gear [10]. More recently, innovations such as carbon fiber, viscoelastic materials, and 3D printing have allowed the creation of lighter, custom-fitted, and biomechanically refined devices [11].

The decision to use this equipment is influenced not only by medical needs but also by regulations, player comfort, appearance, and, in some cases, superstition or media attention. High-profile examples of elite players returning to the field with visible protection have helped shape public opinion and clinical practice [12]. Nevertheless, psychological acceptance, cultural attitudes, and even esthetic considerations continue to play a decisive role in whether athletes consistently use protective devices, underscoring that safety must be evaluated not only biomechanically but also behaviorally.

Although the use of protective gear has become more common, research on its historical development, materials, and design features is still scattered [13]. There is also no clear agreement on how effective specific devices are, especially when it comes to preventing repeat injuries or long-term problems.

This review aims to provide a comprehensive and multidisciplinary overview of protective equipment in football, focusing on its historical development, material innovations, current applications, and regulatory context.

## 2. Material and Methods

### 2.1. Identification of the Research Question

This work is a narrative review aimed at summarizing and critically discussing the evolution and use of protective equipment in football.

### 2.2. Search Strategy

An initial limited search of PubMed/MEDLINE was undertaken to identify articles on the topic and then index terms to develop a full search strategy were used. The marked heterogeneity in study design and outcomes prevented a quantitative synthesis of the data; therefore, a descriptive review approach was adopted. Bibliographic searches on PubMed/Medline, Scopus, Embase and Web of Science have been conducted to identify studies published from 2000 to 2024, using combinations of MeSH terms and free-text keywords related to “Protective Equipment”, “Football”, “Soccer”, “Injury Prevention”, “Shin Guards”, “Headgear”, “Mouthguards”, “Protective Eyewear”, and “Goalkeeper Gloves”. The year 2000 was chosen as the lower cut-off because it coincides with the introduction of modern materials (carbon fiber composites, advanced polymers) and the first scientific reports on protective equipment in football. The bibliographies of the selected articles have been manually examined to identify additional relevant references.

### 2.3. Inclusion Criteria

Studies were included if they involved football/soccer players at any competitive level (professional, semi-professional, amateur; both male and female) and directly analyzed the use, efficacy, or design of protective equipment in matches, training, or laboratory settings. The search was limited to studies published in English, with no restrictions regarding gender or age. When sports-specific literature on football was unavailable, studies from other disciplines addressing similar protective equipment with potential applications to football were considered.

### 2.4. Exclusion Criteria

Studies were excluded if they did not involve football players or focused exclusively on other sports or addressed protective devices only marginally; moreover, studies that were conducted on animals or cadavers, or that provided purely theoretical/technical descriptions without practical application to football.

### 2.5. Data Extraction and Synthesis

All titles and abstracts were independently screened by two authors (M.V. and S.P.), with disagreements resolved by discussion. Full-text articles meeting the inclusion criteria were categorized by device type. Key information extracted included: study design, participant characteristics, type of equipment, outcome measures, and main findings.

Given the narrative nature of the review, data were summarized qualitatively under thematic domains. No formal risk-of-bias or quantitative synthesis was conducted, consistent with narrative review methodology.

### 2.6. Ethical Considerations

This review did not involve human participants or new data collection; therefore, ethics committee approval was not required.

## 3. Historical Overview of Protective Equipment in Football

In the early days of football, protective equipment was virtually nonexistent. Leather shin pads or rudimentary bandages were used sporadically and without regulation. A major turning point came in the late 20th century, when FIFA mandated shin guards as compulsory equipment, marking the first standardized approach to injury prevention in the sport. This regulation not only enhanced safety but also opened the way to innovation in design, comfort, and material engineering.

From a medical perspective, these developments paralleled advances in sports traumatology and rehabilitation. As understanding of musculoskeletal and craniofacial injuries improved, protective devices began to serve not only as preventive tools but also as essential components of safe return-to-play protocols. Facial masks and headgear were progressively introduced [14], often following specific injury cases among elite players. These high-visibility examples accelerated both clinical acceptance and cultural normalization of protective gear in professional football.

Goalkeeper gloves have also evolved remarkably, from simple leather mittens to highly engineered tools with grip-enhancing surfaces, padded zones, and finger-saving spines [15].

Overall, the historical trajectory of protective equipment in football reflects a dynamic interplay between cultural attitudes, technological progress, medical knowledge, and regulatory decisions [16,17,18]. Items once dismissed as unnecessary or stigmatized are now increasingly recognized as essential for safety, comfort, and even performance.

## 4. Typology and Function of Protective Gear

**Figure 1:**
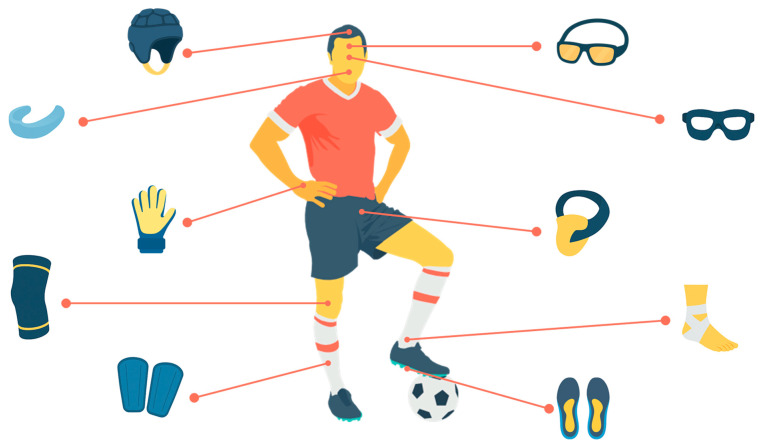
Overview of protective equipment in football. Schematic illustration of the main protective devices used in football, arranged around a central generic player. Clockwise from top left: headgear for impact absorption, sports goggles (standard and wraparound models), facial mask (as used for nasal or orbital fractures), protective cup, ankle brace, custom foot orthotics, shin guards, knee sleeve, goalkeeper glove and mouthguard. The illustration was created with the assistance of ChatGPT (OpenAI, GPT-5 model; https://chat.openai.com, accessed on 5 August 2025).

Protective equipment in football can be broadly divided into two categories [19]. The first includes competition-approved devices, comprising mandatory gear such as shin guards, as well as optional items subject to referee approval, including facial masks, headgear, and protective goggles. The second category refers to auxiliary or supportive devices, such as braces, taping, or insoles, which are frequently used in training, rehabilitation, or prevention programs but are not generally permitted or recognized as protective equipment in official competitions. In addition, certain items (e.g., rigid knee pads, groin protectors) are rarely allowed in matches due to regulatory restrictions or impracticality (Figure 1, Table 1). Despite their widespread use, most of these devices lack standardized performance testing in football [19]. This regulatory gap stands in contrast with other sports, where reproducible laboratory protocols are routinely used to certify protective efficacy.

### 4.1. Shin Guards

They aim to reduce the risk of tibial contusions and fractures resulting from tackles and high-impact collisions, distributing the forces from an impact across a wider area [7]. Modern shin guards are typically constructed from lightweight, impact-resistant materials such as polypropylene, polyurethane, carbon fiber, or composites incorporating foam padding. Variants include slip-in models, ankle-guard extensions, and anatomically contoured designs [20]. Though their efficacy in preventing major fractures is debated, they reduce soft tissue trauma and are widely accepted by players at all levels [7].

Despite being the only mandatory protective device in football, the literature provides limited real-world evidence on their injury-preventive efficacy. Most data come from laboratory impact tests rather than field-based epidemiological studies [21]. There is a need for standardized testing protocols reflecting match conditions, as well as comparative analyses across materials and player levels. Future work should also investigate how comfort and compliance influence actual protection.

### 4.2. Face Masks

Facial masks are increasingly used to enable a safe return to play following midface trauma such as nasal, orbital, orbital, or zygomatic fractures [22]. They reduce the risk of reinjury by protecting the injured bone during the healing process, by transferring the impact forces to the surrounding area. These devices are typically custom-made from facial scans or impressions, using materials such as thermoplastics, carbon fiber, or polycarbonate to combine strength with lightness [23]. A good sports mask needs to balance protection with a wide visual field, comfort, and breathability. Still, players often report issues such as reduced peripheral vision, discomfort during long matches, and the time needed to adapt mentally. In hot or high-intensity situations, sweat buildup and poor ventilation can also make the mask harder to tolerate [24,25]. The spread of 3D printing has notably transformed mask production, allowing for highly accurate fits and faster manufacturing times [26].

Clinical data validating the protective function of facial masks remain limited to case reports and small biomechanical trials. The effects on the visual field, thermoregulation, and player performance are poorly quantified. Future interdisciplinary research combining clinical follow-up, material testing, and player feedback is essential to develop validated safety standards [8].

### 4.3. Headgear

Padded headbands and caps are most often seen in youth leagues or worn by goalkeepers to reduce the forces of head-to-head or head-to-ground impacts [16]. They are generally made from closed-cell foams designed to absorb and dissipate energy. Recent consensus documents, such as those emerging from 2023 youth sports safety initiatives, highlight both the potential role and the current limitations of headgear in pediatric populations [27]. Consequently, their adoption in professional football is minimal, partly due to esthetic concerns and a lack of regulatory endorsement. Beyond biomechanical limitations, headgear adoption is also hindered by cultural and psychological barriers, as some players perceive it as stigmatizing or esthetically undesirable, factors that may outweigh its protective potential [28].

Current evidence remains inconclusive regarding headgear efficacy [12,29]. While some data suggest partial attenuation of linear acceleration, there is no conclusive evidence that it prevents concussion. Research should integrate wearable sensor data, controlled trials, and longitudinal follow-up to assess their real-world benefit, particularly in youth and female athletes.

### 4.4. Goalkeeper Gloves

Although primarily performance-oriented, goalkeeper gloves offer considerable protective benefits, especially against finger hyperextension and wrist injuries [30]. The evidence supporting their role in preventing finger fractures is modest [31]. Modern gloves feature segmented padding, grip-enhancing surfaces, and internal spines or splints to provide finger support [32]. Emerging designs tailored to female goalkeepers have also gained attention, considering anatomical differences and hand size variability. Materials such as latex composites and breathable mesh fabrics offer both functionality and comfort [33].

Scientific validation of glove design is minimal [30,31]. Most developments rely on empirical testing or professional feedback rather than biomechanical studies. Comparative research on grip technology, fatigue reduction, and gender-specific hand morphology would provide stronger evidence for performance and protection optimization.

### 4.5. Mouthguards

Mouthguards are occasionally used by players, particularly those with dental vulnerabilities or a history of orofacial trauma [34]. While not mandated, they can offer adequate protection against tooth fractures and soft tissue injuries following accidental elbow or ball impacts [35]. Custom-fitted designs made of thermoplastic materials provide improved comfort and retention compared to boil-and-bite models [36]. Recent advances include attempts to integrate micro-sensors within mouthguards to measure head impact forces or monitor hydration status, although their application in football remains experimental [37].

Despite widespread recommendations in high-impact sports, mouthguards remain neglected in football due to limited perceived risk and poor usability [36]. Studies linking mouthguard use to head or facial injury rates are needed, along with innovations that improve comfort and aeration without compromising protection.

### 4.6. Glasses

Sports goggles are worn by athletes with refractive errors who cannot tolerate contact lenses or have specific ocular conditions [38]. Unlike conventional eyeglasses, these goggles are shatterproof and equipped with an elastic strap, ensuring stability and safety during high-intensity play. Today, goggles are particularly encouraged in youth categories, where eye trauma and ball-to-face contact are more frequent [39]. In the professional scene, goggles are rarely used. Despite these advantages, fogging, discomfort, and stigma continue to limit widespread adoption, highlighting once again the importance of user-centered design and cultural acceptance.

Although symbolically significant, sports goggles in football lack systematic evaluation. Comparative studies assessing player-reported comfort, visual clarity, and functional confidence when using sports goggles versus contact lenses would also provide valuable insights, particularly for athletes with previous ocular conditions.

### 4.7. Groin Protectors

Protective cups are rarely used in football, as direct impact to the genital area is relatively uncommon. However, they may be considered in specific positions, such as goalkeepers, or during recovery from prior trauma. Comfort concerns and movement restriction limit their widespread adoption, although tailored designs exist. Some sources such as the Cleveland Clinic report that groin protectors as athletic cups can “lower the risk of injuries” but these claims are actually not supported by systematic scientific validation [40]. In American football, protective cups have traditionally been part of the standard equipment—often integrated into jockstraps—particularly at youth levels [41]. Nevertheless, their actual effectiveness in preventing groin injuries is not substantiated by rigorous, sport-specific studies. Future research should clarify their actual protective efficacy in football, as anecdotal use persists without robust biomechanical or epidemiological validation.

### 4.8. Ankle Braces

Ankle braces are frequently used in football, particularly for players with a history of ankle sprains or ligament injuries. Their function is primarily stabilization rather than impact absorption, aiming to reduce the risk of recurrent injuries during training and competition [42]. A systematic review and meta-analysis showed that lace-up braces significantly reduced ankle injury incidence, especially in players with a previous sprain history [43]. Compared to taping, bracing offers equal or greater protection and maintains consistent support over time [44]. Nevertheless, regulatory ambiguity persists, as braces are variably permitted in official competitions, underscoring the need for consensus guidelines balancing safety with freedom of movement.

### 4.9. Knee Braces

Knee braces, including functional braces and compression sleeves, are often used after anterior cruciate ligament reconstruction or meniscal surgery. While some studies indicate they may improve proprioception and provide psychological reassurance, others find no measurable biomechanical or injury-preventive advantage [45]. Despite this, their use persists in elite football as part of a comprehensive rehabilitation protocol. From a psychological perspective, however, many athletes report increased confidence when using functional braces, suggesting that their benefit may extend beyond biomechanics into the domain of mental readiness and adherence [46].

Evidence suggests mechanical benefits primarily in players with previous injuries, while prophylactic use in healthy athletes remains controversial. Integrating biomechanics, proprioception assessment, and player adherence studies would clarify their role in personalized protection strategies.

### 4.10. Foot Orthoses

Custom foot orthoses are employed to address biomechanical anomalies such as overpronation or leg length discrepancy [47,48]. They are supposed to improve plantar load distribition thus to reduce overuse injuries but it seems to be more of a belief and a marketing strategy not supported by real scientific data. Furthermore, to date, it is still very unclear how custom insoles actually work compared to prefabricated models, which—when indicated—may represent a more cost-effective alternative without compromising clinical outcomes [49,50,51].

## 5. Materials and Design Innovations

The development of protective equipment in football has progressed in parallel with innovations in materials science and design. A comparative overview of the main materials used in football protective equipment is provided in Table 2, summarizing their typical applications, mechanical characteristics, advantages and disadvantages. Despite these advances, football lacks standardized protocols to evaluate the mechanical performance of protective materials. While drop tests, force-distribution mapping, and durability assays are widely used in sports such as American football or hockey, no uniform criteria currently exist for football gear [13].

What once consisted of basic leather pads or cloth wrappings has now evolved into sophisticated devices engineered for both protection and performance. This transformation reflects a growing understanding that safety must not come at the expense of mobility, comfort, or user adherence [52].

One of the most significant innovations in recent decades has been the use of carbon fiber composites [53]. Carbon fiber composites are now widely used in facial masks and shin guards for their high stiffness-to-weight ratio, allowing impact dispersion without restricting movement, an important aspect for athletes requiring both protection and agility. Thermoplastic materials (e.g., polycarbonate, polypropylene) enable anatomical customization, improving fit and comfort during prolonged activity and facilitating safe return-to-play after injury [54]. The combination of 3D scanning and additive manufacturing has enabled the rapid production of custom-fit devices with unprecedented precision [55]. Meanwhile, viscoelastic foams and gel inserts enhance comfort and shock absorption, reducing soft-tissue trauma and potentially influencing proprioceptive feedback, an emerging determinant in neuromuscular injury prevention [56].

The most transformative shift in recent years has come from the integration of digital and wearable technologies. Indeed, embedding smart sensors into protective equipment enables real-time monitoring of impact forces, load distribution, and hydration status [13]. These data can complement clinical assessments, supporting injury prevention, rehabilitation, and return-to-play decisions. Modern protective equipment is therefore evolving from passive protection toward active, data-informed systems that integrate safety, performance optimization, and individualized athlete care [13].

## 6. Regulation and Guidelines

The use of protective equipment in football is primarily governed by the Laws of the Game, established by the International Football Association Board (IFAB) with enforcement delegated to match officials and tournament organizers [10]. Among all forms of gear, only shin guards are explicitly mandated, as outlined in Law 4, required to be covered by socks, made of suitable materials, and provide a reasonable degree of protection. However, the law lacks specifications regarding size, impact attenuation, or biomechanical standards, resulting in substantial variability among commercial products.

Beyond shin guards, all other protective devices are approved on a case-by-case basis, provided they pose no danger to the player or others [10]. In practice, referees retain discretionary power, which can lead to inconsistent implementation across competitions. FIFA and UEFA guidelines suggest transparency for face masks and color neutrality, but no formal certification process exists. In contrast, sports such as American football, ice hockey, and baseball have long implemented detailed performance standards—often through NOCSAE or ASTM protocols—that define minimum impact attenuation thresholds, penetration resistance, or durability requirements [57]. Even within Europe, where CE marking regulates medical devices, most football equipment does not fall under medical-grade certification, creating a regulatory gray zone [58]. No dedicated ISO standards exist to certify football gear, further limiting international harmonization and consumer protection.

Pediatric and amateur leagues may adopt stricter or more lenient approaches, especially regarding headgear use for concussion prevention, which remains a topic of debate [59]. In such contexts, national federations or school-based associations may recommend or even require specific equipment, often based on evolving evidence and local policy. This variability is especially evident in pediatric and youth settings, where concussion awareness campaigns have encouraged headgear use in some jurisdictions, while others refrain due to insufficient scientific evidence [60]. Such inconsistency complicates both parental decision-making and industry innovation.

The absence of sport-specific performance standards and certification pathways limits both consumer protection and evidence-based practice in football. From a sports medicine standpoint, this gap complicates clinical recommendations on return-to-play and protective use following injury. Future frameworks should integrate biomechanical validation, clinical efficacy data, and regulatory oversight, bridging sports engineering and medical expertise.

## 7. Media Cases and Athlete Perspectives

Iconic athletes and media exposure have long shaped the public perception of protective gear in football. While initially stigmatized or viewed as a sign of vulnerability, visible protective equipment has increasingly become normalized through high-profile examples.

**Figure 2:**
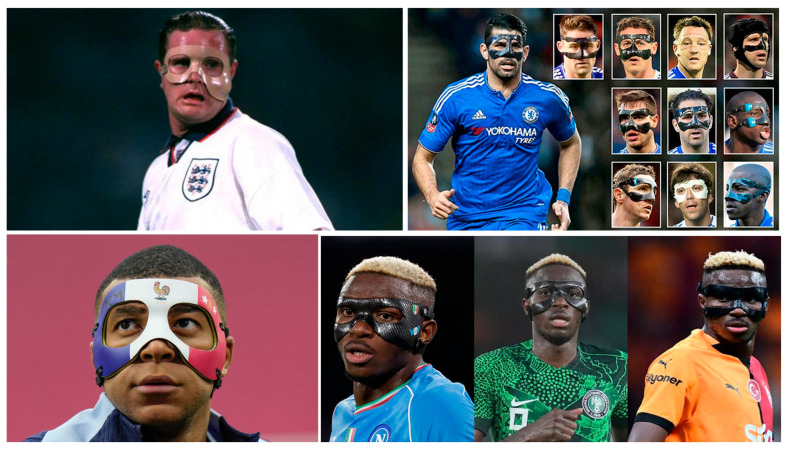
Evolution and personalization of facial protective masks in football. Top left: From the early and rudimentary transparent mask worn by Paul Gascoigne in the 1990s, to the widespread use of carbon fiber masks in the 2000s and 2010s, the adoption of facial protection in football has evolved both materially and culturally. Top right: Humorously references a group of Chelsea FC players who returned to play wearing masks after facial injuries, so numerous that they were described as forming “a squad within the squad.” Bottom left: Kylian Mbappé initially trained with a French tricolor mask following a nasal fracture at Euro 2024; however, UEFA regulations prohibited national colors or symbolic markings on field equipment, requiring him to switch to a plain version during official matches. Bottom right: Victor Osimhen has continued wearing his mask post-recovery, making it part of his public identity. All images used in Figure 2 are reproduced properly credited to their original sources: Top left: Paul Gascoigne wearing a protective face mask—BBC Sport, “Mbappe injury—11 images of famous players in face masks.”, retrieved from [bbc.com/sport/football/articles/c10014gvj2jo] (accessed on 10 October 2025) referencing Getty Images. Top right: Diego Costa among the Chelsea “masked men XI”—Daily Mail Online, “Diego Costa to wear protective mask against Newcastle to complete Chelsea’s masked men XI,” 12 February 2016, [https://www.dailymail.co.uk/sport/football/article-3444824/Diego-Costa-wear-protective-mask-against-Newcastle-complete-Chelsea-masked-men-XI.html] (accessed on 10 October 2025). Bottom left: Kylian Mbappé with tricolored mask during Euro 2024 training—AP News, “Masked Mbappé trains for Netherlands match at Euro 2024, coach optimistic he’ll play.” [apnews.com/article/mbappe-euro2024-france-525b593071f9734544f72310573e57d8] (accessed on 10 October 2025) referencing Getty Images. Bottom right: Victor Osimhen wearing a protective mask—images sourced from: Distractify (“Why does Osimhen wear a mask?”) referencing Getty Images, [distractify.com/p/why-does-osimhen-wear-a-mask] (accessed on 10 October 2025); The Sporting News article: [https://www.sportingnews.com/us/soccer/news/victor-osimhen-mask-face-explained-nigeria/dd89916277034b2e50cd0ef0] (accessed on 10 October 2025); Mardin Life: https://www.mardinlife.com/gundem/galatasaray-in-yildiz-golcusu-osimhen-neden-maske-takiyor (accessed on 10 October 2025).

Historically, one of the first notable instances of facial protection was seen in Paul Gascoigne, who wore a crude face mask after suffering a cheekbone fracture in the early 1990s. At the time, such devices were rare and often improvised. His case drew attention not only for its novelty but also for the limitations and esthetic awkwardness of early protective designs. In recent years, Antonio Rüdiger has become emblematic of the modern protective mask. Following facial fractures, he wore a black carbon-fiber mask while playing in the German Bundesliga, the English Premier League, and Spain’s La Liga—demonstrating its functional utility across different football cultures. His aggressive and effective performances helped destigmatize such equipment. Victor Osimhen represents a fascinating shift: after recovering from multiple facial fractures and orbital reconstruction surgery, the Nigerian striker continued wearing his custom mask permanently, reportedly for both comfort and psychological security—but also as a stylistic element, now part of his on-field identity (Figure 2) [61].

These cases highlight that the adoption of protective gear is not only determined by medical indication but also by psychological reassurance, cultural acceptance, and media exposure. Players often report that protective devices provide confidence and reduce anxiety about re-injury, while others abandon them due to discomfort or perceived stigma. Validated psychological tools, such as the Competitive State Anxiety Inventory-2 (CSAI-2), could be applied in future studies to quantify the impact of protective equipment on confidence, perceived vulnerability, and adherence [62].

Petr Čech, following a life-threatening skull fracture in 2006, adopted padded headgear for the rest of his career [63]. His case brought long-term cranial protection into the global spotlight. Similarly, Cristian Chivu, after undergoing cranial surgery, used custom headgear throughout his final seasons in Italian Serie A, helping to further normalize protective headgear. These high-profile examples also exposed the lack of uniform regulatory guidance, as headgear remained approved only at referee discretion, despite growing clinical and cultural recognition of its potential role.

More recently, attention has turned to the increasing number of players who routinely wear wrist strapping, even without an apparent medical need. This trend has been particularly evident during the 2023–2024 season, when several FC Barcelona players were regularly seen using visible wrist protection during competitive matches [64].

One of the most distinctive and medically motivated examples of protective gear use in elite football is that of Edgar Davids, the Dutch midfielder who competed at the highest level in the 1990s and early 2000s with clubs including Ajax, FC Internazionale, Juventus, AC Milan, and Tottenham Hotspur. Davids became an iconic figure on the pitch for his energetic style of play and his constant use of protective sports goggles in every official match. This choice was driven by medical necessity rather than esthetics, as he was diagnosed with glaucoma in 1999 and subsequently underwent eye surgery. Following the procedure, he developed photophobia and increased susceptibility to eye trauma. To continue playing safely, he adopted custom-made, shatterproof goggles with photochromic lenses and a secure elastic strap, ensuring both visibility and protection. To date, he remains one of the only professional footballers at the top level to wear such protective eyewear on the field routinely [65]. His case also underscores how medical necessity can intersect with personal branding, as the goggles became not only a safety device but also a defining part of his athletic image. This blurring of medical utility and stylistic identity has since influenced how protective equipment is perceived by both fans and athletes.

Collectively, these cases demonstrate that protective gear in football serves purposes that extend well beyond injury prevention. Devices may provide psychological reinforcement, become embedded in personal or cultural identity, or even act as fashion statements. The visibility of such equipment in elite competitions continues to shape public perception, drive market trends, and influence adherence across all levels of the game.

## 8. Practical Applications

The synthesis presented in this review has several practical implications for daily football practice.

- Clinicians can use this evidence to support the prescription of protective equipment during rehabilitation and return-to-play, particularly in cases of fractures, ligament injuries, and facial trauma.
- Coaches and trainers can better understand which devices are competition-approved and which are auxiliary, allowing them to guide athletes appropriately in both training and matches.
- Players can benefit from increased awareness of the protective, psychological, and cultural value of equipment, helping them to make informed decisions and to improve adherence.
- Medical staff should be aware that materials such as viscoelastic foams or latex grips degrade rapidly with sweat exposure and repeated washing, which can compromise protection and comfort. Providing athletes with clear guidance on replacement intervals and hygiene practices could significantly improve adherence and safety.
- Regulators and governing bodies may use these insights to harmonize approval criteria, standardize testing methods, and encourage innovation while ensuring player safety.
- Industry stakeholders can leverage insights from sports engineering to integrate wearable sensors into protective gear transforming them into multipurpose tools for both safety and performance monitoring. Although currently experimental, such innovations may soon reach practical application in football.

## 9. Challenges and Future Directions

A major unresolved challenge is the absence of standardized testing across product categories. Unlike American football or hockey, where impact attenuation and drop tests certify helmets and padding, football equipment lacks reproducible performance benchmarks. Establishing such standards would provide objective safety data, guide regulatory approval, and enhance player trust [66].

Durability and hygiene remain underexplored. Sweat absorption, cleaning cycles, and environmental exposure can degrade material performance, yet systematic protocols for hygiene testing and replacement intervals are lacking. Integrating these factors into design and regulation would improve both safety and adherence [67,68]. Protective devices must balance safety with comfort, esthetics, and psychological readiness. Future research should incorporate validated tools—such as athlete-reported questionnaires and adherence monitoring systems—to objectively assess comfort and user compliance [69,70].

Looking ahead, adding wearable technology to protective equipment could open new possibilities [71]. Wearable technologies embedded in shin guards, headgear, or mouthguards could enable real-time monitoring of impacts, motion, and physiological status. Despite promising prototypes, barriers such as miniaturization, durability, and lack of standardized protocols must be overcome before widespread adoption [72,73,74]. Future research will need to address these challenges before wearable sensors can be routinely embedded into protective gear and used for both injury prevention and performance monitoring [75].

In parallel, FIFA and IFAB have introduced broader initiatives, including concussion protocols and discussions on limiting headers in youth football, that complement protective gear and highlight the need for integrated medical, regulatory, and technological strategies [76,77]. Such measures complement protective equipment and highlight the need for integrated approaches that combine regulatory action, medical oversight, and technological innovation.

Finally, current evidence is limited by small case series and laboratory surrogates, with a lack of longitudinal studies assessing reinjury rates, career impact, and long-term musculoskeletal or neurological outcomes. Addressing these gaps will be essential for translating protective equipment from anecdotal use into evidence-based clinical and regulatory practice.

## 10. Conclusions

In football, protective equipment has progressed from rudimentary improvisations to highly engineered devices shaped by advances in materials science, biomechanics, sports medicine, and digital technologies such as 3D printing. Currently, shin guards remain the only mandatory gear, while other devices play important but varied roles: facial masks enable safe return-to-play after midface fractures and are increasingly accepted; headgear is mainly used in youth football, though evidence for concussion prevention remains inconclusive; and orthoses and braces are widely applied in clinical practice despite heterogeneous scientific support. Adoption of protective equipment is not determined solely by medical necessity but also by psychological reassurance, cultural acceptance, and practical considerations such as comfort, esthetics, durability, and hygiene. These factors strongly influence adherence and should be systematically evaluated alongside biomechanical performance.

Yet critical challenges persist. Football still lacks standardized testing protocols, clear regulatory frameworks, and longitudinal evidence on long-term outcomes. Gaps are particularly evident in youth populations and in emerging categories of equipment, where inconsistent guidelines hinder both clinical recommendations and innovation. As the game becomes faster and more physically demanding, the need for effective protective solutions will continue to grow. Meeting this demand will require stronger collaboration between clinicians, researchers, governing bodies, manufacturers, and players. Future progress will rely on integrating biomechanics, materials science, clinical evidence, and regulatory clarity into a unified approach, ensuring that protective equipment enhances both safety and performance in modern football.

## Figures and Tables

**Table 1:** Protective equipment in football: indications, evidence of effectiveness, and limitations.

Device	Indications for Use	Effectiveness (Evidence)	Limitations	Notes/Gaps
**Shin guards**	Mandatory in all competitions; protection against tibial fractures and contusions	Observational studies show reduced tibial injury incidence	Quality varies; lack of standardized performance criteria	Mandatory by FIFA
**Face masks**	Facial fractures, nasal injuries; used to allow faster return-to-play	Mild evidence of higher protection and earlier RTP	Limited evidence base; referee approval discretionary	Case-by-case approval
**Headgear**	Protection against superficial head injuries; post-concussion use	Mixed evidence, no clear reduction in concussions	Comfort/adherence issues; stigma; no uniform standards	Optional use
**Goalkeeper gloves**	Finger and hand protection; improved grip	Widespread use; limited evidence on reduction in finger injuries	Grip materials degrade; hygiene concerns	Universally used
**Mouthguards**	Dental/facial trauma prevention	Effective in reducing dental injuries	Discomfort; poor adherence among players	Not mandated in football
**Sports goggles**	Vision correction, eye protection (players with impaired vision)	Reduce ocular trauma risk; allow participation	Fogging, discomfort, stigma	Permitted but anecdotal
**Groin protectors**	Rare in football; used in cases with prior injury or added risk	Effective in other sports (e.g., American football); no football-specific data	Limited acceptance; often considered cumbersome	Not adopted in professional football
**Ankle braces**	Secondary prevention after sprain/ligament injury	Studies in other sports suggest reduced reinjury rates	May restrict motion; not always permitted in competition	Often classified as therapeutic
**Knee braces**	Post-ligament injury support; RTP facilitation	Mild evidence of reducing reinjury in other sports	Bulky; adherence issues; limited football-specific research	Often classified as therapeutic
**Foot orthoses**	Correction of biomechanical deficits; overuse injury prevention	Some evidence in reducing stress injuries/plantar fasciitis	Debate if truly “protective”; regulation varies	Not controlled

**Table 2:** Comparative analysis of materials in football protective equipment.

Material	Typical Applications	Mechanical Properties (Qualitative)	Advantages	Disadvantages	Hygiene/Washability
Polypropylene	Shin guards, protective shells	Lightweight, stiff, moderate impact absorption	Low cost, durable, widely available	Limited comfort, less conformable	Easy to clean, resistant to sweat
Carbon fiber composites	Facial masks, rigid braces	High strength-to-weight ratio, rigid, excellent load dispersion	Superior protection, custom molding possible	High cost, brittle under extreme loads	Easy to sanitize, resistant
Thermoplastics (e.g., EVA, TPU)	Shin guards, padding inserts	Flexible, moldable, moderate durability	Good balance between cost and comfort, versatile	Deforms over time, less durable	Washable, moderate resistance to wear
Viscoelastic foams	Headgear, protective padding	High impact absorption, soft, compressible, flexible	High comfort, excellent shock attenuation	Poor long-term durability, absorbs sweat	Harder to clean, retains moisture

## Data Availability

No new data were created or analyzed in this study. Data sharing is not applicable to this article.
